# Supplementary material for: Delta Opioid Peptide Targets Brain Microvascular Endothelial Cells Reducing Apoptosis to Relieve Hypoxia-Ischemic/Reperfusion Injury
Source: Pharmaceutics. 2022 Dec 23;15(1):46. doi: 10.3390/pharmaceutics15010046 (PMC9861451; doi:10.3390/pharmaceutics15010046)
Supplement: Supplementary file 1 [file pharmaceutics-15-00046-s001.zip › pharmaceutics-2023725-supplementary.pdf]

pharmaceutics

MDPI

Supplementary Materials: Delta Opioid Peptide Targets Brain Microvascular Endothelial Cells Reducing Apoptosis to Relieve Hypoxia-Ischemic/Reperfusion Injury

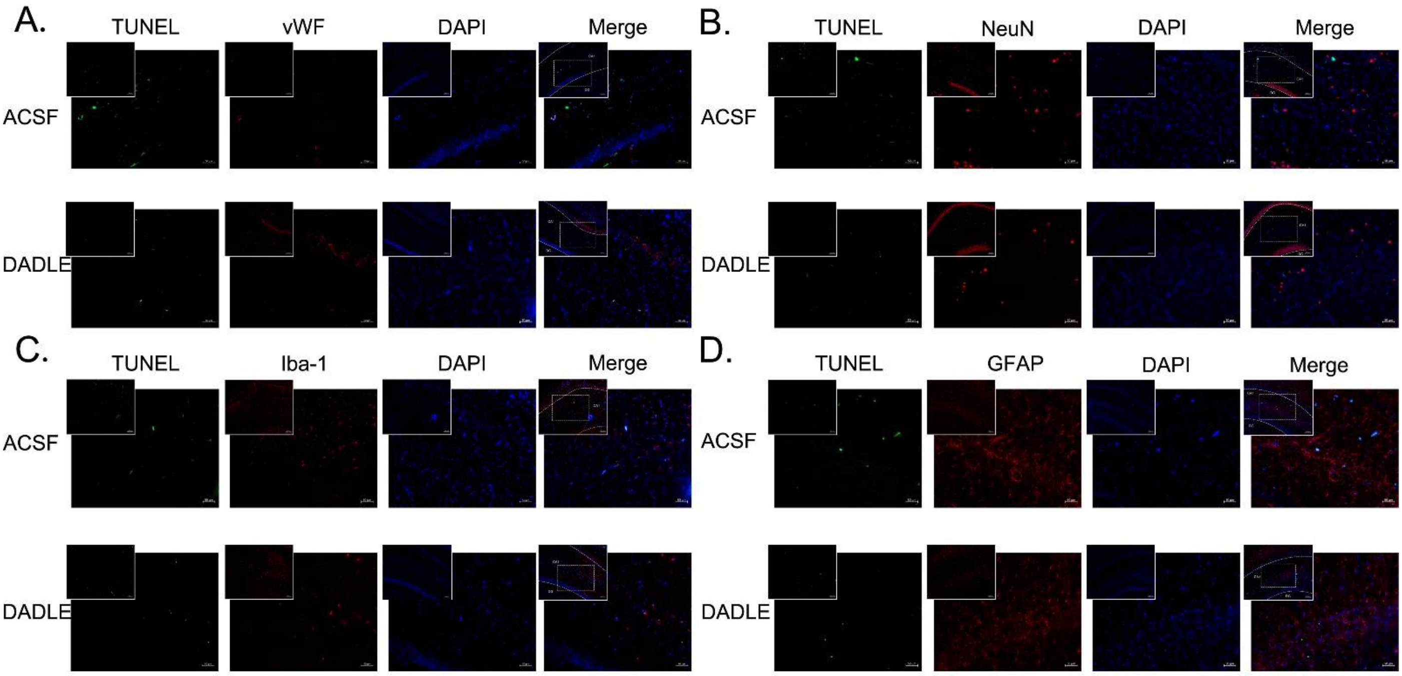

Figure S1. MCAO/R-induced cellular apoptosis in the CA1 area of the hippocampus mainly co-localized with vascular endothelial cells, but not neuron, microglia nor astroglia, and DADLE (5 nmol/10 μL) pretreatment decreased endothelial apoptosis at 72 h after surgery. (A-D) TUNEL-positive cells were stained green. DAPI represented nuclei and showed as blue. Red fluorescence signals respectively represented vWF indicating vascular endothelial cells (A), NeuN indicating neuron (B), Iba-1 indicating microglia (C) and GFAP indicating astroglia (D). n=3 in all the groups.

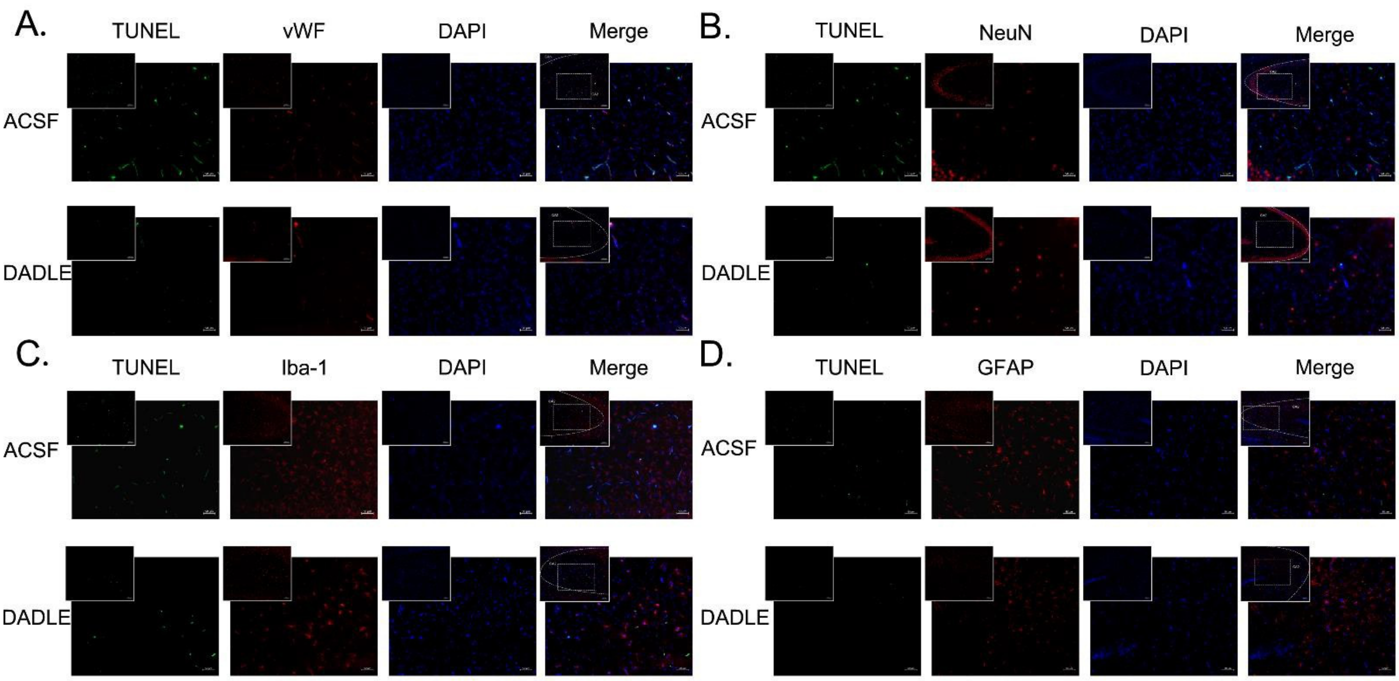

Figure S2. MCAO/R-induced cellular apoptosis in the CA2 area of the hippocampus mainly co-localized with vascular endothelial cells, but not neuron, microglia nor astroglia, and DADLE (5 nmol/10 μL) pretreatment decreased endothelial apoptosis at 72 h after surgery. (A-D) TUNEL-positive cells were stained green. DAPI represented nuclei and showed as blue. Red fluorescence signals respectively represented vWF indicating vascular endothelial cells (A), NeuN indicating neuron (B), Iba-1 indicating microglia (C) and GFAP indicating astroglia (D). n=3 in all the groups.

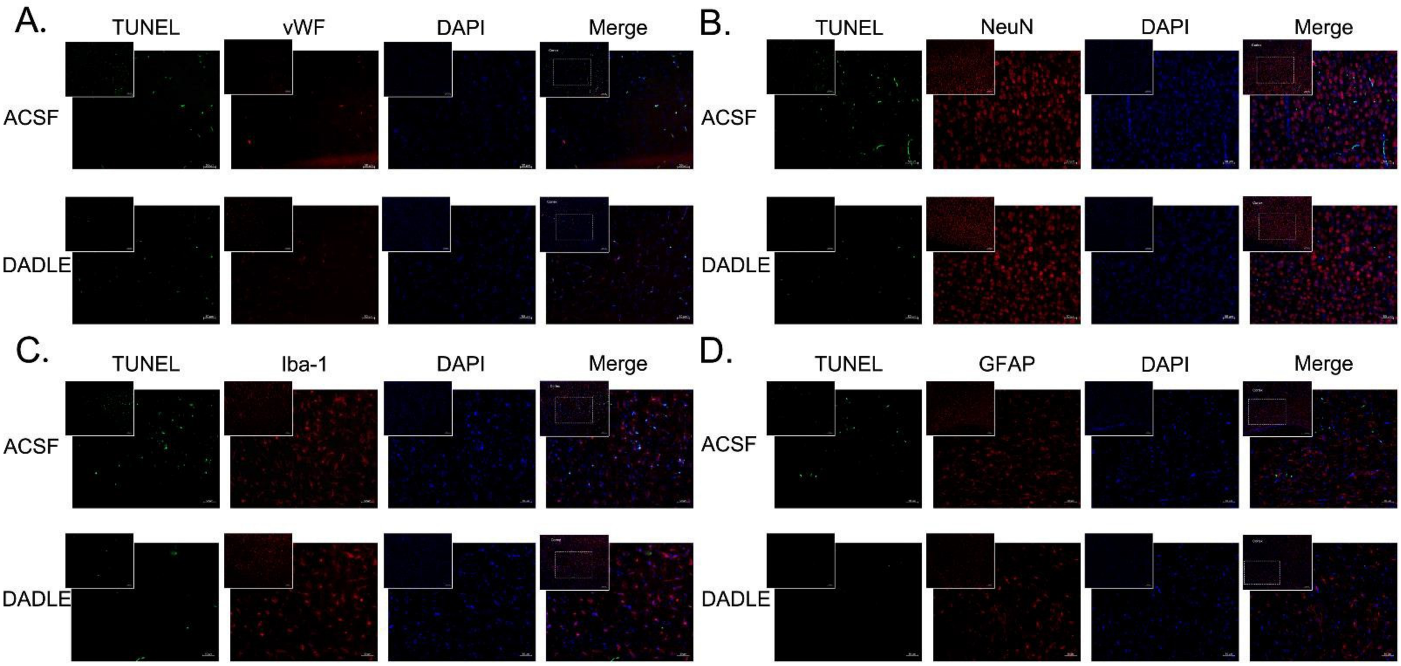

Figure S3. MCAO/R-induced cellular apoptosis in the cortex mainly co-localized with vascular endothelial cells, but not neuron, microglia nor astroglia, and DADLE (5 nmol/10 μL) pretreatment decreased endothelial apoptosis at 72 h after surgery. (A-D) TUNEL-positive cells were stained green. DAPI represented nuclei and showed as blue. Red fluorescence signals respectively represented vWF indicating vascular endothelial cells (A), NeuN indicating neuron (B), Iba-1 indicating microglia (C) and GFAP indicating astroglia (D). n=3 in all the groups.

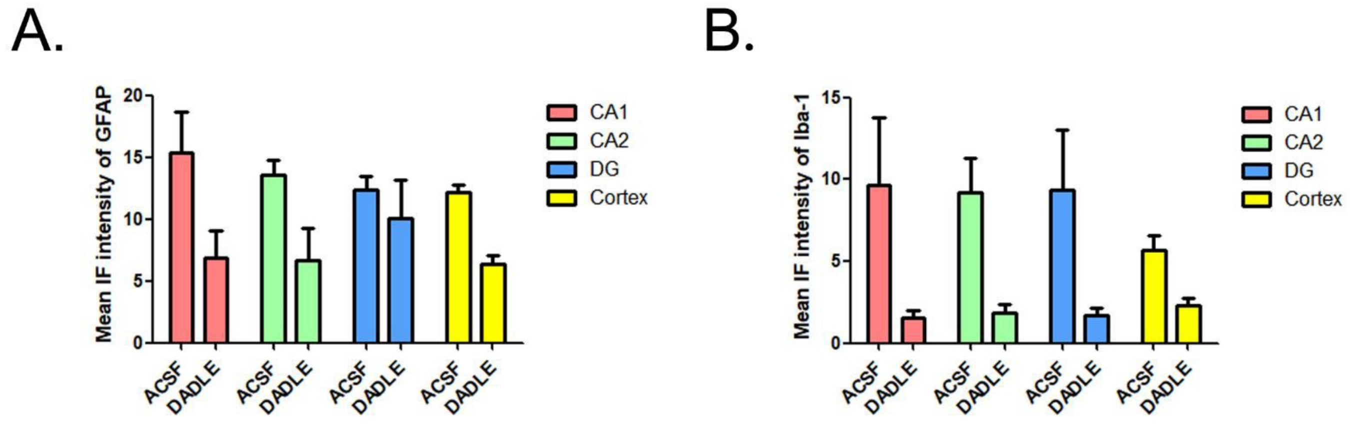

Figure S4. Statistical analysis of the fluorescence intensity changes of GFAP-(A) and Iba-1-(B) positive signals between the ACSF- and DADLE-administered MCAO/R groups within the CA1, CA2 and DG of the hippocampus or cortex area. Bars represent the mean ± S.E.M. Every group contained data from three rats.

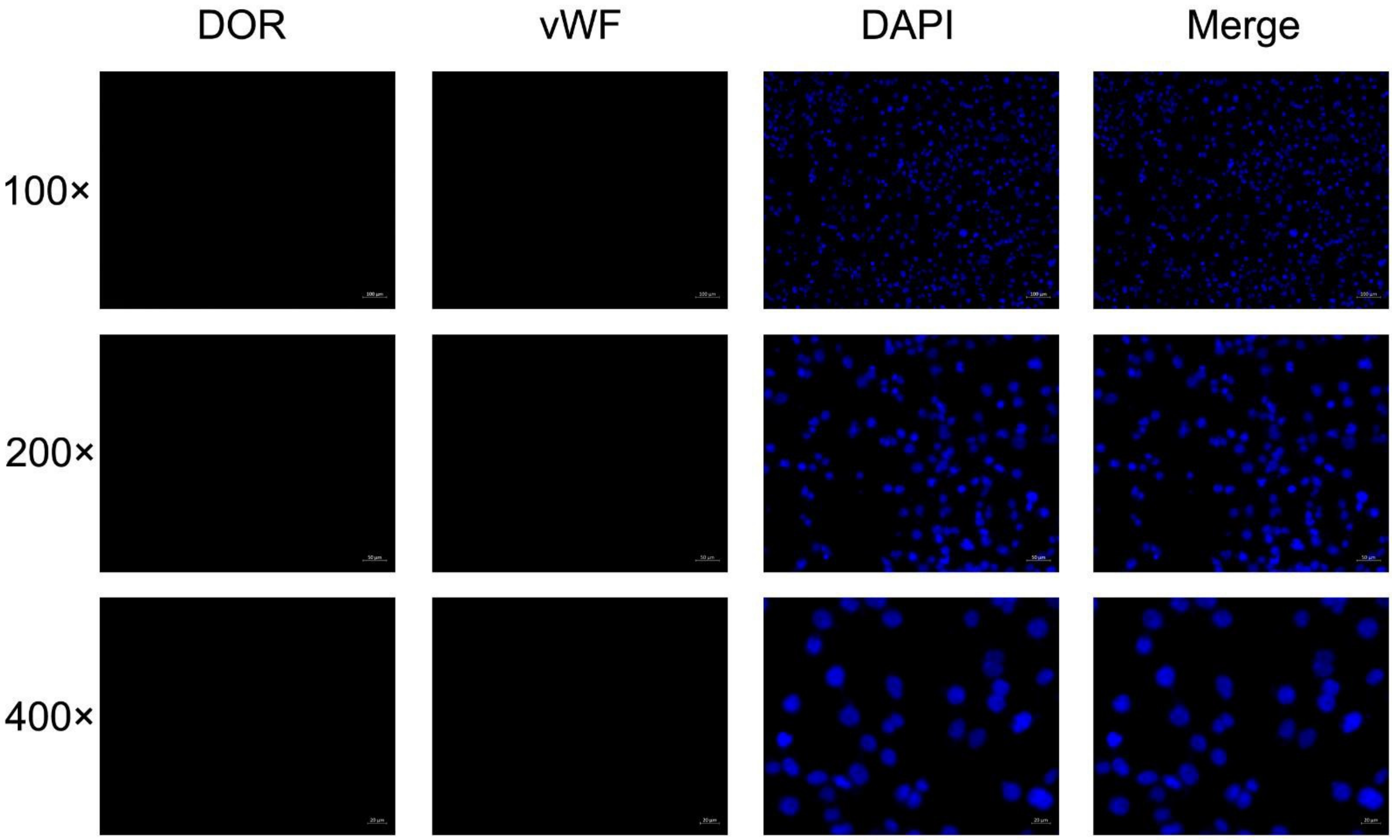

Figure S5. The HEK293 cells do not express δORs nor vWF. (A) Typical immunofluorescence pictures showed the expression of δORs (Green) and vWF (Red) on the BMECs. DAPI represented the nuclei and showed as blue. Individual experiments were repeated three times.
